# Complete Genome Sequence and Annotation of Malacoplasma iowae Type Strain 695, Generated Using PacBio Sequencing

**DOI:** 10.1128/mra.00490-22

**Published:** 2023-01-04

**Authors:** Mostafa Ghanem, Amro Hashish, Dhiraj Chundru, Mohamed El-Gazzar

**Affiliations:** a Department of Veterinary Medicine, Virginia-Maryland College of Veterinary Medicine, University of Maryland, College Park, Maryland, USA; b Department of Veterinary Diagnostic and Production Animal Medicine, College of Veterinary Medicine, Iowa State University, Ames, Iowa, USA; c National Laboratory for Veterinary Quality Control on Poultry Production, Animal Health Research Institute, Agriculture Research Center, Giza, Egypt; Indiana University, Bloomington

## Abstract

Malacoplasma iowae, previously known as “Mycoplasma iowae,” is associated with embryo mortality, reduced hatchability, and leg abnormalities in turkeys, leading to considerable economic losses. Here, we report the complete and annotated genome sequence of Malacoplasma iowae type strain 695.

## ANNOUNCEMENT

Malacoplasma iowae type strain 695 (MI_695_OSU_1.1) was acquired from David Ley of North Carolina State University and originally purchased from the American Type Culture Collection. The frozen aliquot was grown in 10 mL of Modified Ortmeyer (M-ORT) broth medium at 37°C (1). After 4 days, the culture was centrifuged at 415 × *g* for 30 min. The pellets were suspended in 200 μL of phosphate-buffered saline (PBS) for DNA extraction using the Genomic-tip 100/G device (Qiagen, Valencia, CA). The extracted DNA was quantified using a NanoDrop 2000 spectrophotometer (Thermo Scientific, Wilmington, DE) and was determined to comprise 533.32 ng/μL. The same pool was measured using a Qubit fluorometer (Thermo Fisher Scientific, USA) and was estimated to comprise 113.2 ng/μL. The fragment size was measured using the TapeStation system (Agilent Technologies, Inc.). The average fragment size was more than 60 kbp. A 20-kb template preparation was performed according to the Pacific Biosciences procedure (2). Briefly, DNA was sheared using a g-TUBE device (Covaris, LLC). Then, fragments of 15 kb to 20 kb were size selected using the BluePippin system (Sage Scientific, Beverly, MA, USA).

The genome of Malacoplasma iowae type strain 695 was sequenced using the SMRTbell standard DNA library and single-molecule real-time (SMRT) technology on the PacBio RS II platform (Pacific Biosciences, Menlo Park, CA) according to the standard manufacturer’s protocol at the University of Delaware DNA Sequencing & Genotyping Center (Newark, DE). The filtered raw reads were assembled to generate one continuous contig without any gaps using the Hierarchical Genome Assembly Process (HGAP) version 3.0 with default parameters, except for the estimated genome size, which was 1.3 Mbp using the SMRT Portal (3). The subread count was 54,182, comprising a total of 491,585,679 bp. The mean subread length and *N*_50_ value were 9,072 bp and 13,241 bp, respectively. The number of adaptor dimers was 0.02%.

The assembled genome of *Malacoplasma iowae* type strain 695 is a single circular chromosome consisting of one contig of 1,315,476 bases, with an average coverage of 310.53× and an overall GC content of 24.64%. There were 43,964 reads, and the *N*_50_ read length was 15,228 bp. The mean read score was 0.68. The average reference consensus concordance was 100%. The assembled genome was checked for overlap, trimmed, and rotated using the Circlator tool (4). An updated version of the assembled genome was annotated using the NCBI Prokaryotic Genome Annotation Pipeline (PGAP) version 6.3.0 (5). The annotated genome had 1,046 protein-coding sequences (CDS), 29 tRNA genes, 3 rRNA genes, 3 noncoding RNAs (ncRNAs), 2 CRISPR arrays, and 8 pseudogenes.

The availability of this complete genome sequence of Malacoplasma iowae, along with its annotation, will broaden our knowledge of the genome structure, functions, and dynamics and improve our understanding of many biological insights, including pathogenic mechanisms, virulence factors, and immune dominant antigens. Additionally, this will help in developing robust genotyping schemes for epidemiological investigations and subsequently, better formulation of prevention, control, and eradication strategies.

### Data availability.

The *Malacoplasma iowae* type strain 695 genome assembly has been deposited at GenBank under accession number CP033512.2. The raw reads have been deposited under SRA accession number SRP384144. The NCBI BioProject accession number is PRJNA503681.
